# Efficient Translation of *Pelargonium line pattern virus* RNAs Relies on a TED-Like 3´-Translational Enhancer that Communicates with the Corresponding 5´-Region through a Long-Distance RNA-RNA Interaction

**DOI:** 10.1371/journal.pone.0152593

**Published:** 2016-04-04

**Authors:** Marta Blanco-Pérez, Miryam Pérez-Cañamás, Leticia Ruiz, Carmen Hernández

**Affiliations:** Instituto de Biología Molecular y Celular de Plantas (Consejo Superior de Investigaciones Científicas-Universidad Politécnica de Valencia), Valencia, Spain; University of British Columbia, CANADA

## Abstract

Cap-independent translational enhancers (CITEs) have been identified at the 3´-terminal regions of distinct plant positive-strand RNA viruses belonging to families *Tombusviridae* and *Luteoviridae*. On the bases of their structural and/or functional requirements, at least six classes of CITEs have been defined whose distribution does not correlate with taxonomy. The so-called TED class has been relatively under-studied and its functionality only confirmed in the case of *Satellite tobacco necrosis virus*, a parasitic subviral agent. The 3´-untranslated region of the monopartite genome of *Pelargonium line pattern virus* (PLPV), the recommended type member of a tentative new genus (*Pelarspovirus*) in the family *Tombusviridae*, was predicted to contain a TED-like CITE. Similar CITEs can be anticipated in some other related viruses though none has been experimentally verified. Here, in the first place, we have performed a reassessment of the structure of the putative PLPV-TED through *in silico* predictions and *in vitro* SHAPE analysis with the full-length PLPV genome, which has indicated that the presumed TED element is larger than previously proposed. The extended conformation of the TED is strongly supported by the pattern of natural sequence variation, thus providing comparative structural evidence in support of the structural data obtained by *in silico* and *in vitro* approaches. Next, we have obtained experimental evidence demonstrating the *in vivo* activity of the PLPV-TED in the genomic (g) RNA, and also in the subgenomic (sg) RNA that the virus produces to express 3´-proximal genes. Besides other structural features, the results have highlighted the key role of long-distance kissing-loop interactions between the 3´-CITE and 5´-proximal hairpins for gRNA and sgRNA translation. Bioassays of CITE mutants have confirmed the importance of the identified 5´-3´ RNA communication for viral infectivity and, moreover, have underlined the strong evolutionary constraints that may operate on genome stretches with both regulatory and coding functions.

## Introduction

Translation of viral products is the first step in the reproductive cycle of eukaryotic plus-strand RNA viruses once they enter a cell and become uncoated. As viruses do not encode their own ribosomes, such a process entirely relies on the translational machinery of the host [1–3]. In eukaryotes, the cytosolic translation apparatus usually recognizes monocystronic mRNAs with a 5´-cap (m^7^GpppN) and a 3´-poly(A) tail, two structures that function synergistically to facilitate translation [4, 5]. The 5´-cap is bound by eukaryotic initiation factor (eIF) 4E, the smallest component of the eIF4F complex. The largest component of this complex, eIF4G, acts as scaffold for assembly of other initiation factors including eIF4A, eIF4B and poly(A)-binding protein (PABP), which in turn binds to the 3´-poly(A) tail. The 43S ribosomal preinitiation complex (comprising the 40S subunit, the eIF2–GTP–Met-tRNAi^Met^ ternary complex, eIF5, eIF3, eIF1 and eIF1A) is then recruited to the mRNA via an eIF3-bridged interaction with eIF4G, and starts scanning in a 5´-to-3´ direction until an initiation codon in a good context is reached. At this point, most of the initiation factors are released, the 60S ribosomal subunit joins forming the 80S ribosome and protein synthesis ensues [6, 7]. During the initiation stage, the simultaneous interaction of eIF4G with eIF4E, that is bound to the 5´-cap, and to PAPB, that is bound to the 3´-poly(A) tail, causes circularization of the mRNA which seems to enhance recruitment and recycling of the 40S ribosomal subunit [8, 9].

Despite the dependence of viruses on the host for translation, many eukaryotic viral mRNAs lack the 5´-cap structure and/or the 3´-poly(A) tail typically found in cellular mRNAs. That is the case for the vast majority of plant plus-strand RNA viruses but, importantly, still they are fully competent for translation. This competence is normally achieved through alternative, non-canonical translation mechanisms [10–12]. One of these mechanisms involves the use of 3´-proximal RNA sequences as cap-independent translational enhancers (CITEs) [12, 13]. So far, at least six classes of CITEs have been defined that differ in their structural and/or functional requirements [14, 15]. No correlation among CITE classes and viral groups can be established suggesting that these regulatory elements have evolved independently and/or have been acquired through modular shuffling via recombination [15–17]. Although the mechanism of action of CITEs is not fully understood, it is generally accepted that they recruit key components of the host translational machinery for subsequent delivery to the 5´-end of the viral mRNA to initiate translation [18]. Two main pieces of evidence supporting this model are: i) the detection of interactions (at least *in vitro*) of CITEs with components of the eIF4F complex (or of its plant isoform, eIFiso4F; [19]) or even directly with ribosomal subunits, and ii) the frequent complementarity between CITEs and 5´-proximal sequences of the corresponding viral RNA that may aid in transfer of the CITE-recruited factor(s) [14, 15]. In addition, genetic evidence for the interaction between a viral 3´-CITE and eIF4E has been obtained in one case [20].

**Fig 1:**
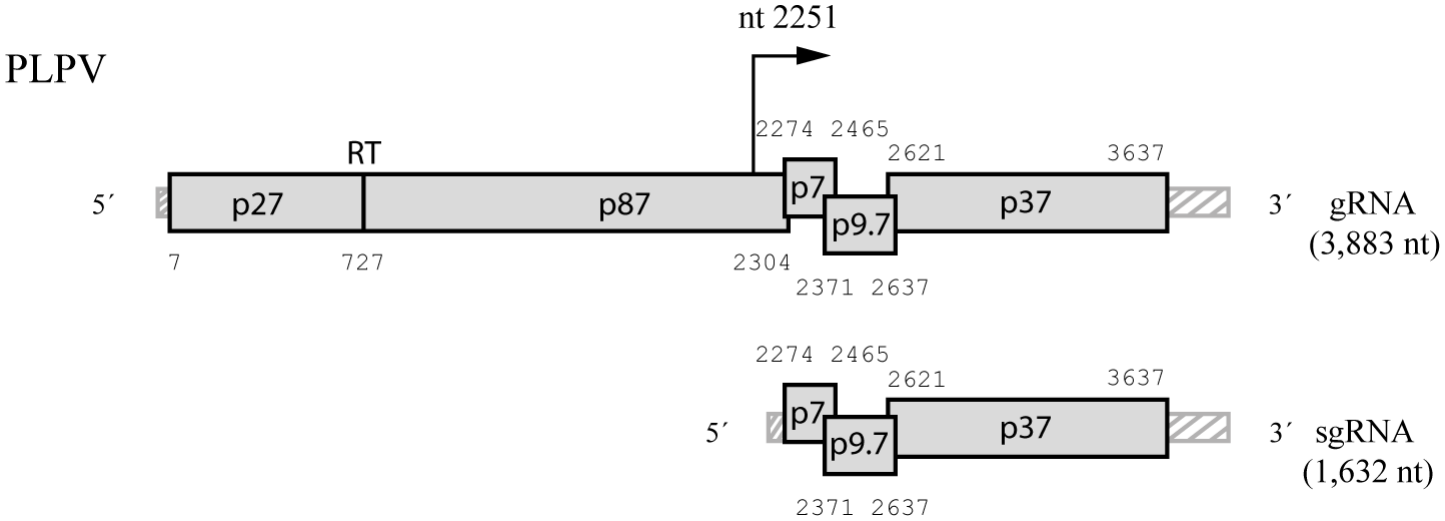
Schematic representation of the PLPV genome. PLPV gRNA contains five ORFs (represented by grey boxes) flanked by 5´- and 3´- UTRs (striped boxes). The viral RNA dependent-RNA polymerase, p87, is the ribosomal read-through (RT) product of p27, an auxiliary replication protein. PLPV sgRNA is tri-cistronic and serves as mRNA for expression of two small movement proteins, p7 and p9.7, and of protein p37, which functions as coat protein and as the suppressor of RNA silencing.

*Pelargonium line pattern virus* (PLPV) possesses a monopartite, plus-strand RNA genome that is encapsidated into icosahedral particles of about 30 nm in diameter. The genomic RNA (gRNA) is composed by 3883 nucleotides (nt) and lacks both a 5´-cap and a 3´-poly(A) tail and contains five open reading frames (ORFs) flanked by an unusually short 5´-untranlated region (5´-UTR) of only 6 nt, and by a 3´-UTR of 246 nt (Fig 1). The two 5´-proximal ORFs encode two proteins involved in replication, p27 and p87 (the viral RNA dependent-RNA polymerase, RdRp). Two centrally located ORFs encode two small movement proteins (MPs), p7 and p9.7, and the 3´-proximal ORF encodes a protein, p37, that has a dual role as coat protein (CP) and as viral suppressor of RNA silencing (VSR) [21–23]. PLPV is a member of large family *Tombusviridae*, which currently encompasses thirteen genera and more than fifty species [24–26], and bears close resemblances with species of genus *Carmovirus* as regards to its genomic organization and protein sequences. However, several traits differentiate PLPV from carmoviruses including: i) the production of a single, tricistronic subgenomic (sg) RNA for expression of 3´-proximal ORFs (Fig 1) in contrast with carmoviruses that generate two sgRNAs for this purpose, ii) the presence of a non-AUG start codon in MP2 gene instead of the canonical AUG found in the corresponding gene of carmoviruses and, iii) the lack of AUGs in any frame between the AUG initiation codons of MP1 and CP genes unlike carmoviruses that harbour 1–8 AUGs in the equivalent region [21, 22]. These molecular features are shared by at least four structurally and phylogenetically related viruses, which has prompted the proposal of their inclusion in a new genus, tentatively named *Pelarspovirus*, within family *Tombusviridae*, with PLPV recommended as the type species [21, 27–29].

Data obtained in recent years have revealed interesting aspects concerning the regulation of gene expression in PLPV. Some of those data enabled us to confirm, as expected from comparative analysis with related viruses (21), that ORFs p27 and p87 are translated directly from the gRNA, the latter via readthrough of the ORF p27 leaky stop codon. The unique viral sgRNA that is produced during infection serves as mRNA for translation of ORFs p7, p9.7 and p37 [21, 22]. Expression of all three genes from the tricistronic sgRNA seems coordinated through leaky-scanning favoured by the suboptimal translational context of the initiating AUG of MP1 gene, the non-canonical start codon (GUG) of MP2 gene and the lack of any AUG triplet between the AUG initiation codons of MP1 and CP genes. Despite these significant advances in understanding the gene expression strategy of PLPV, little is know about how PLPV (g and sg) RNAs attract the translation machinery in the absence of a 5´- cap structure and a 3´-poly(A) tail. *In silico* analyses allowed prediction of a potential CITE in the viral 3´-UTR that would have structural resemblances with the so-called translation enhancer domain (TED) of *Satellite tobacco necrosis virus* (STNV), the first 3´-CITE discovered [15, 30, 31]. Significant data on the mode of action of STNV-TED are available. It has been reported to interact with eIF4F and eIFiso4F in wheat germ extracts (WGE) through their cap binding subunits, eIF4E and eIFiso4E [32]. The way in which the bound factors are repositioned at the 5´-end has not been ascertained, but several hypotheses have been advanced. It was proposed that the global tertiary structure of the viral RNA could bring the 5´- and 3´-UTRs of STNV RNA into close proximity [33]. Also, that communication between both terminal regions would be mediated by proteins [32] or could be facilitated by stretches of sequence complementarity [31, 34]. However, attempts to prove the relevance of potential 5´-3´ base-pairing(s) were unfruitful [34], and thus the mechanism underlying the transfer of the factors recruited by STNV-TED remains an open question.

Apart from STNV-TED, no other TED-like CITE has been experimentally investigated. In this work, we have focused our attention on the TED-like CITE presumably present at the 3´-UTR of PLPV. A fourfold objective has been pursued: i) to re-asses the structure of the putative PLPV-TED through *in silico*, *in vitro* and *in vivo* approaches, ii) to determine whether the predicted element really works as a CITE and, if so, iii) to obtain insights into the RNA features that influence its activity and, iv) to verify the relevance of CITE-mediated translation for viral infectivity. The results have revealed that the structure of the presumed PLPV-TED is larger than anticipated. Moreover, they have confirmed that the element functions as a CITE *in vivo* (although not *in vitro*) in both the gRNA and the sgRNA and that its activity depends on the establishment of a long-range RNA-RNA interaction with a hairpin of the corresponding 5´-region. Data on some other structural requirements for CITE function have also been obtained. In addition, bioassay of CITE mutants in plants have corroborated that maintenance of the base-pairing interaction between the 5´-region and 3´-CITE is critical for virus viability and have suggested that the regulatory and coding functions of the 5´-partner(s) impose important evolutionary constraints.

## Material and Methods

### DNA constructs

Two pUC18-based PLPV wild-type (wt) constructs containing cDNAs encompassing full-length gRNA and sgRNA sequences, respectively, plus an added 5´-proximal T7 RNA promoter have been described previously [22, 35]. These plasmids were used as templates to generate a set of mutant constructs bearing deletions in the PLPV 3´-UTR. The deletion mutants were generated through PCR using *Pfu Ultra* DNA polymerase (Stratagene) and different pairs of primers (one complementary and one homologous to distinct regions of the PLPV sequence in each pair) that harbored appropriate restriction sites to facilitate subsequent cloning steps. PCR products (comprising the cloning vector, pUC18, fused to a PLPV 5´- region at one side and to a PLPV 3´-region at the other side) were eluted after agarose electrophoresis, digested with the corresponding restriction enzyme(s), self-ligated and cloned following standard protocols.

PLPV gRNA- or sgRNA-derived reporter constructs were generated through replacement of specific viral genome segments by Firefly luciferase (Fluc) gene. To facilitate such replacement, proper restriction sites (*Nco*I and *Pst*I at the 5´- and 3´-side, respectively) were introduced in both genomic and subgenomic T7-driven constructs. Deletions in some reporter constructs were made as indicated above and nucleotide replacements in other constructs were engineered by PCR with the Quick Change Site-Directed Mutagenesis kit (Stratagene) and specific oligonucleotide pairs.

Each mutant construct was verified by DNA sequencing with an ABI PRISM DNA sequencer 377 (Perkin-Elmer). The type and position of the modifications present in each construct is indicated in the Figures depicting the mutants.

### *In vitro* transcription and translation

Uncapped transcripts were synthesized *in vitro* with T7 RNA polymerase (Thermo Scientific) from PLPV (genomic and subgenomic)-derived constructs linearized with *Sma*I [22, 35]. Capping of some RNAs was performed with the ScriptCap m^7^G Capping System according to the manufacterer´s protocol (Epicentre Biotech.). The transcripts were used for *in vitro* translation experiments with WGE (Promega) including [^14^C]leucine to label *de novo* synthesized proteins as described previously [22]. Translation products were separated by SDS-polyacrylamide gel electrophoresis and labelled proteins were quantified with the aid of a PhosphorImager (Fujifilm FLA-5100, GE Healthcare).

### Protoplast preparation, inoculations and measurement of luciferase activity

Protoplasts (0.5 x 10^6^), prepared from *Nicotiana benthamiana* leaves as previously described (36), were inoculated with 20 μg of uncapped transcripts generated from each Fluc reporter construct along with 1 μg of capped transcripts synthesized from a Renilla luciferase (Rluc) reporter construct (internal control) [20] using polyethylene glycol. Protoplasts were harvested 3 h later and lysed with 1× passive lysis buffer (Promega). Luciferase activity was measured with a Dual-Luciferase Reporter Assay System (Promega) employing the GloMax-Multi Detection System (Promega). All constructs were assayed three times in totally independent protoplast preparations. For each construct, translation efficiency corresponding to the mean and standard error of the mean from the three replicates was calculated.

### Inoculation of plants

Uncapped transcripts from PLPV gRNA constructs were used to mechanically inoculate *Nicotiana benthamiana* plants (two carborundum dusted-leaves per plant employing approximately 2 μg of transcript per leave). Plants were maintained under greenhouse conditions (16 h days at 24°C, 8 h nights at 20°C) and leaf material was harvested 15 days after inoculation for further analysis. For serial passage experiments, *Chenopodium quinoa* plants were mechanically inoculated with transcripts (passage 0) and sap from infected material was used as inoculum for serial viral transfer to new *C*. *quinoa* plants at 10 day intervals.

### RNA extraction, Northern blot hybridization and RT-PCR amplification

Total RNA preparations of *N*. *benthamiana* and *C*. *quinoa* leaves were obtained by phenol extraction and LiCl precipitation [36]. In addition, for RNA stability assays, protoplasts were collected at 1 and 3 h after inoculation and total RNA was extracted using NucleoZOL reagent according the manufacturers instructions (Macherey-Nagel). Northern blot analysis was performed with a ^32^P-radioactive RNA probe, derived from PLPV p37 gene or from the Fluc gene, as previously described [23]. In serial passage experiments, total RNA extracts from virus-infected leaf material were subjected to RT-PCR amplification with SuperScript II One-Step RT-PCR System (Invitrogen) using virus-specific primers. Purified PCR products were either directly sequenced or cloned into pTZ57R/T vector (Thermo Scientific) prior to sequencing.

### RNA secondary structure predictions and SHAPE structure probing

Secondary structure predictions were performed using the Mfold program version 4.6 (www.bioinfo.rpi.edu/applications/mfold) [37]. In addition, full-length PLPV gRNA transcripts were employed to assay RNA structure in the TED and flanking regions using SHAPE (selective 2´-hydroxyl acylation analyzed by primer extension) [38]. RNA (500 ng per reaction) was heated at 95°C for 3 min and snap-cooled on ice for 2 min. The RNA was then renatured in SHAPE folding buffer (100 mM HEPES pH 8.0, 6 mM MgCl_2_, 100 mM NaCl) for 5 min at 37°C. Next, N-methylisatoic anhydride (NMIA) dissolved in DMSO (final concentration of NMIA corresponding to 6 mM) was added while only DMSO was added to the control. The reaction mixtures were incubated at 37°C for 15 min and the RNA was recovered by ethanol precipitation and washed three times with ethanol 70%. Another set of snap-cooling, renaturing and NMIA treatment conditions [39] were also used for comparison in parallel assays. As the results of both approaches were identical, only those obtained with the protocol described above will be shown in the Results section. Oligonucleotides were 5´-end radiolabeled with γ-[^32^P]-ATP and T4 polynucleotide kinase and primer extension reactions were performed using Superscript III reverse transcriptase (Invitrogen) as previously reported [38]. An oligonucleotide complementary to positions 3685–3703 was employed. DNA products of the reverse transcription reactions were resuspended in deoinized formamide and subjected to electrophoresis through 6% denaturing polyacrylamide gel. Radioactive bands were visualized by autoradiography or with a PhosphorImager (Fujifilm FLA-5100). Sequencing ladders were generated by Sanger sequencing method using unmodified RNA, the same 5´-end labelled oligonucleotides used for the modified RNA and Superscript III reverse transcriptase.

## Results

### Potential TED-like CITE in the 3´-UTR of PLPV and prediction of similar CITEs in other proposed pelarspoviruses

**Fig 2:**
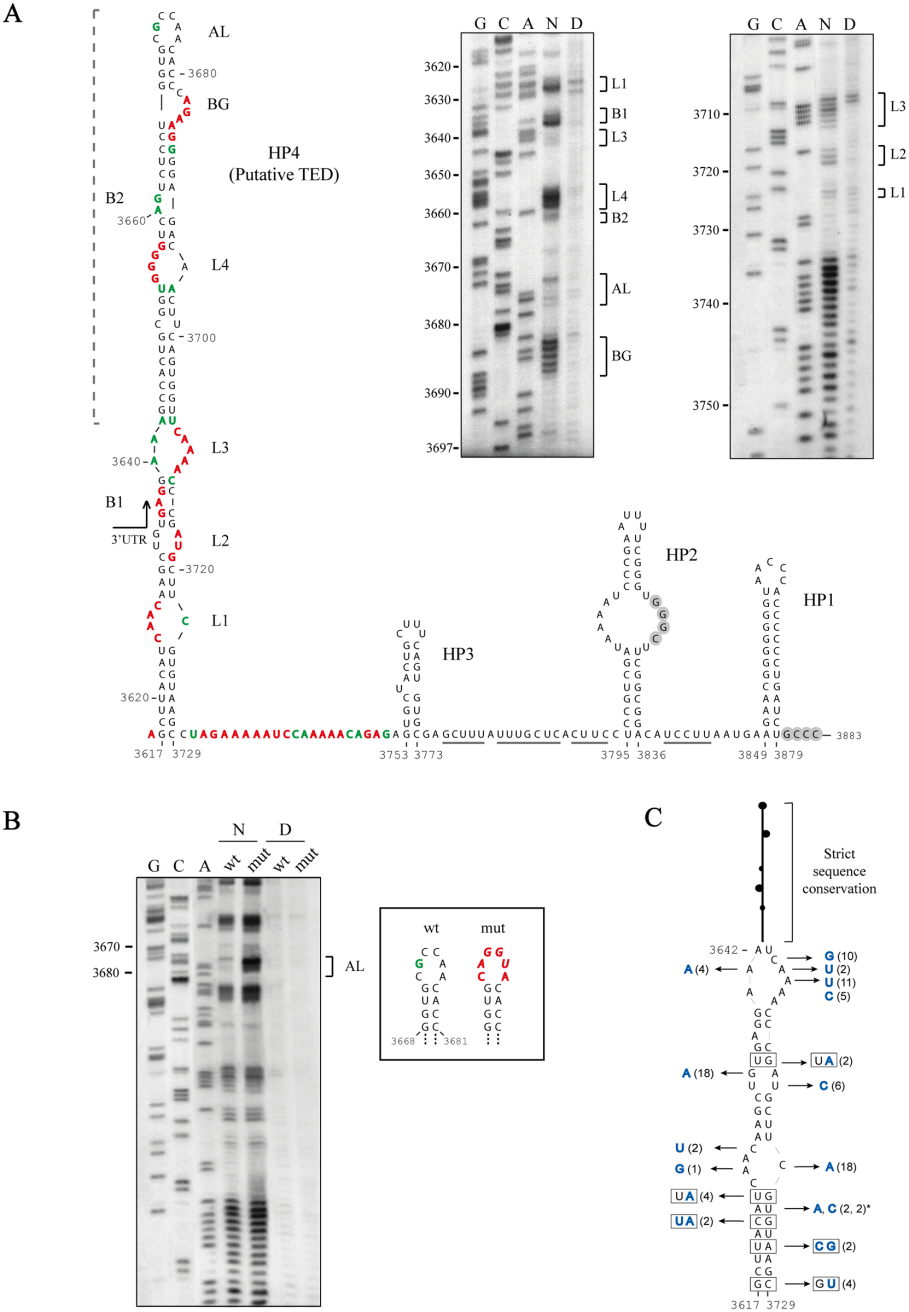
Examination of the structure of the 3´-terminal region of PLPV through *in silico*, *in vitro* and comparative structural analyses. (A) Secondary structure of the 3´-terminal region of PLPV (bottom). The represented structure embraces the 3´-terminal sequence of the p37 gene and the entire 3´ UTR (arrow), contains several hairpins (HP1 to HP4) and corresponds to the optimal one according to Mfold predictions using the entire PLPV gRNA. Nucleotides likely involved in an interaction that presumably acts as repressor of minus-strand synthesis are within grey circles. Underlined nucleotides are predicted to base-pair with a segment of the gRNA out of the 3´-terminal region. HP4, that might be a CITE of the TED class, shows a SHAPE-derived flexibility profile (autoradiographs at the top) consistent with the Mfold predictions. In autoradiographs of SHAPE analysis, the lanes have been labelled as: N, for the NMIA treated RNA, D, for the DMSO treated RNA, G, C and A, for sequencing ladders. Residues in the putative TED and flanking region with high and medium reactivity to NMIA are denoted with red and green colors, respectively, whereas those with low or no reactivity to the reagent are in black. Bulges (B) and loops (L) of HP4 have been numbered on the secondary structure representation to facilitate comparison with SHAPE profile. The apical loop and a lateral bulge of the upper part of HP4 have been labelled as AL and BG, respectively. The region of HP4 previously proposed as putative TED-like CITE (15) is indicated by a square bracket with discontinuous line at the left side of the structure. (B) SHAPE profiles across a portion of the 3´-terminal region of the PLPV wild-type (wt) gRNA and a mutated (mut) gRNA carrying nucleotide replacements (in italics) in the apical loop of HP4. Strong NMIA reactivity of the apical loop in the mutant is observed when compared with the wt molecule. This result is in agreement with the involvement of the loop in RNA-RNA interaction as previously proposed [15]. The lanes have been labelled as in panel A and the sequencing ladders have been performed on the wt template. (C) Distribution of natural sequence heterogeneity along HP4. Twenty natural PLPV variants [43] were included in the analysis. Nucleotide replacements are in blue and the number of variants with a given mutation is shown in parentheses. Co-variations and mutations resulting in conversion of canonical Watson-Crick to wobble base pairs, or vice versa, in stems are boxed. Only two minority mutations affecting residue U3723 (marked by an asterisk), could lead to slight destabilization of a stem.

The most likely structure of the 3´-terminal region of PLPV is depicted in Fig 2. Such structure was retrieved as the optimal one (i.e., that of lowest free energy) when the entire PLPV gRNA was subjected to *in silico* structural analysis with the Mfold program [37]. The obtained conformation was composed by several hairpins (HP1 to HP4 in Fig 2A). As found in some members of family *Tombusviridae* (genera *Carmovirus* and *Umbravirus*), the 3´-proximal HP (HP1 in Fig 2A) most likely works as core promoter for transcription of complementary strands [40, 41] whereas the 3′-penultimate HP (HP2 in Fig 2A) contains a large symmetrical internal loop whose 3´-side is expected to act, through a base-pairing interaction with the four 3’-terminal nucleotides, as repressor of minus-strand synthesis [42, 43]. The potential TED-like CITE would correspond to the upper part of the 5´-proximal HP (HP4 in Fig 2A) according to previous proposals [15, 44]. However, these proposals were mostly based on structural *in silico* analysis using just the 3´-UTR which precluded prediction of the entire HP4 since this hairpin embraces nucleotides not only of the 3´-UTR, but also of the p37 gene (Fig 2A). The *in silico* analysis performed here with the complete gRNA molecule suggested that the putative PLPV-TED could be larger than previously anticipated as it could correspond to the whole HP4.

The validity of the predicted structure of HP4 and flanking region was assessed by SHAPE, an approach that interrogates local backbone flexibility at single-nucleotide level [38]. Briefly, an acydilating agent, usually NMIA, is used to modify 2´-OH groups of unconstrained nucleotides in an RNA molecule. Modified sites are detected as stops in primer extension assays, allowing researcher to draw an RNA structural map on the basis of SHAPE reactivity. SHAPE probing of the putative TED and surrounding sequences in the full-length PLPV gRNA was highly consistent with the *in silico* structural predictions (Fig 2A). Despite the high level of correlation between the computer-assisted folding of the presumed TED and that deduced from SHAPE assay, a striking difference concerned the predicted apical loop of the structure that showed poor reactivity to NMIA. This observation suggested that such a loop may be involved in RNA-RNA interaction(s) with other region(s) of the gRNA molecule, as previously proposed [15]. In agreement with this view, the loop became fully accessible to NMIA modification when four out of its six nucleotides were mutated in the gRNA molecule, whereas the reactivity of other HP4 residues remained unaltered (Fig 2B). This observation nicely supported both the proposed HP4 structure and the participation of the apical loop in RNA-RNA interaction(s).

Natural variation can be extremely useful in substantiating the biological soundness of RNA conformations obtained by *in silico* and *in vitro* methods [45, 46]. The major assumption underlying the analysis of sequence variants in this context is that mutations should tend to preserve functionally important RNA structures. A previous study on the molecular diversity of PLPV isolates from different origins showed the presence of polymorphic positions along distinct regions of the viral genome [43]. We examined the distribution of the sequence heterogeneity found in twenty PLPV molecules -those reported in reference [43]- on the structure of HP4 as revealed by *in silico* and *in vitro* approaches (Fig 2C). The sequence of the upper part of this HP was strictly conserved in all PLPV variants, as previously noted [43], but numerous nucleotide replacements were found mapping to its lower part (Fig 2C). The conformation of this portion of the HP was highly supported by natural mutations because a large proportion of nucleotide substitutions were located in loops and, moreover, because of the presence of co-variations or compensatory mutations (converting canonical into G:U wobble pairs or vice versa) that maintain base-pairing in stems. These observations provided comparative structural evidence in support of the secondary structure of HP4 derived from computer-assisted predictions and SHAPE data.

**Fig 3:**
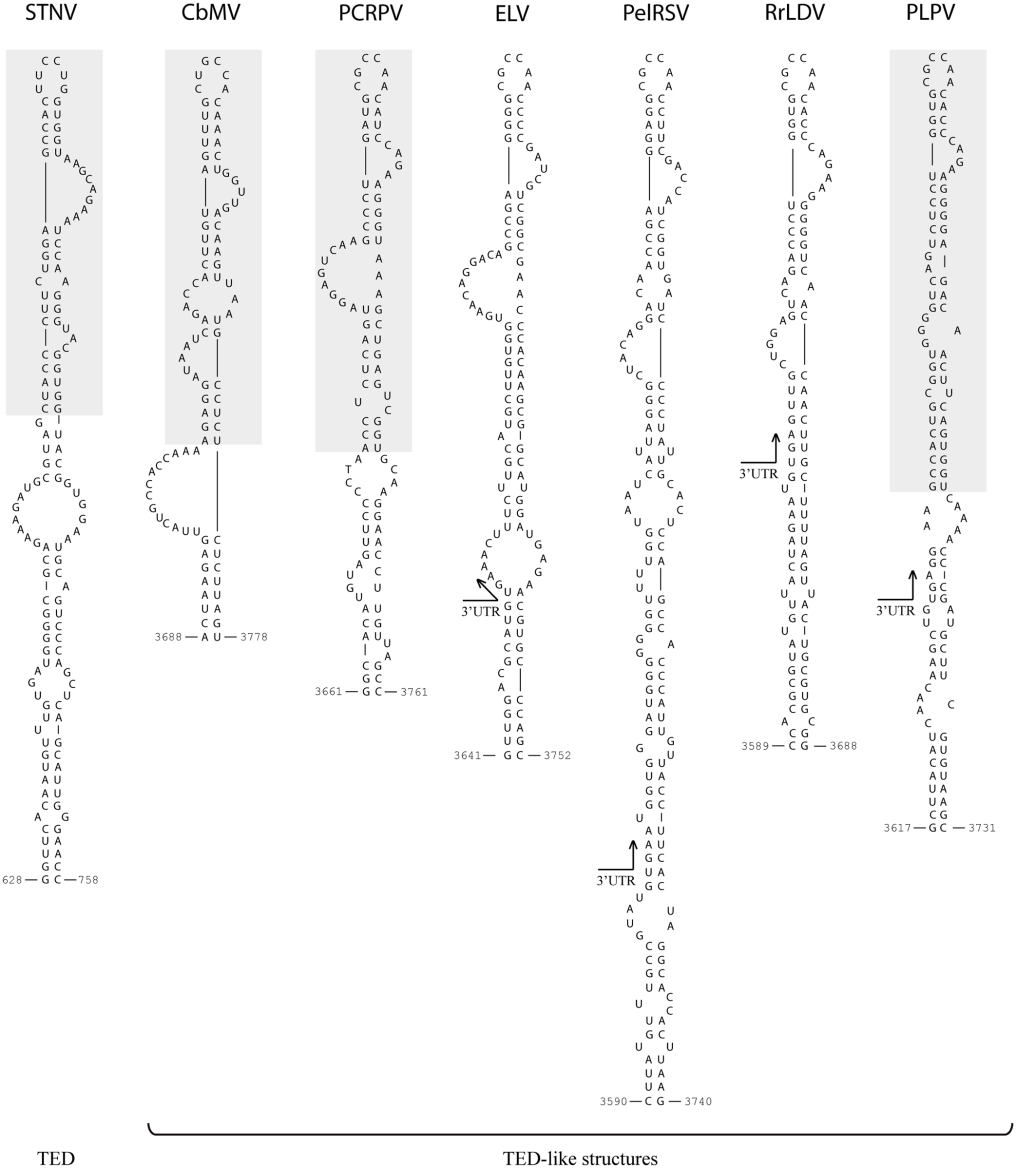
Proposed structures of STNV-TED and of TED-like CITEs predicted in several viruses. Virus acronyms: *Satellite tobacco necrosis virus* (STNV), *Calibrachoa mottle virus* (CbMV), *Pelargonium chlorotic ring pattern virus* (PCRPV), *Elderberry latent virus* (ELV), *Pelargonium ring spot virus* (PelRSV), *Rosa rugosa leaf distortion virus* (RrLDV) and *Pelargonium line pattern virus* (PLPV). CbMV is a member of genus *Carmovirus* and PCRPV, ELV, PelRSV, RrLDV and PLPV are recommended to be included in the tentative new genus *Pelarspovirus*, all within family *Tombusviridae*. The region corresponding to the previously proposed STNV-TED as well as those corresponding to the previously proposed TED-like CITEs of CbMV, PCRPV and PLPV [15] are shown on a grey background.

In the light of the structure deduced for PLPV HP4 (the putative TED) by *in silico* and *in vitro* approaches and by comparative analysis of natural virus variants, a reassessment of the STNV-TED structure was performed by using the complete STNV RNA for Mfold predictions. Similarly to that found for the potential TED-like CITE of PLPV, a more extended conformation than that previously proposed (15) was obtained for STNV-TED (Fig 3). In addition to PLPV, TED-like CITEs have been predicted in *Pelargonium chlorotic ring pattern virus* (PCRPV), a tentative member of genus *Pelarspovirus*, and in *Calibrachoa mottle virus* (CbMV), a member of genus *Carmovirus* [15], and can be also anticipated in the other three tentative pelarspoviruses, *Elderberry latent virus* (ELV), *Rosa rugosa latent virus* (RrLDV) and *Pelargonium ringspot virus* (PelRSV) (Fig 3). Mfold analyses with the full-length gRNAs of these viral agents revealed long hairpin configurations for the putative TED-like CITEs of all of them (Fig 3), thus giving further support to the enlarged structure of the presumed translational element.

The putative TED of PLPV shows clear structural resemblances with STNV-TED and also some differences (Fig 3). Prominent among the latter is the dissimilar sequence of the 6 nt-apical loop, an observation that also applies for the 6-nt apical loop of the predicted TED-like CITE of CbMV (Fig 3). In contrast, the sequence of the apical loop (5´-CGCCAA-3´) as well as that of the closing base pair (G:C) was identical in the predicted TED-like CITEs of all pelarspoviruses, thereby further extending the number of common traits between members of the proposed new genus. It should be also mentioned that the sequence of the apical loop of TED-like structures of all pelarspoviruses as well as of CbMV fits the consensus motif (5´-YGCCA-3´) that, as previously noted [15], is conserved in the apical loop of CITEs of PTE (*Panicum mosaic virus*-like translational enhancer) and I-shaped classes of carmoviruses as well as in the apical loop of a T-shaped CITE of an umbravirus.

### *In vivo* translation assays confirm the functionality of the predicted PLPV TED-like CITE

**Fig 4:**
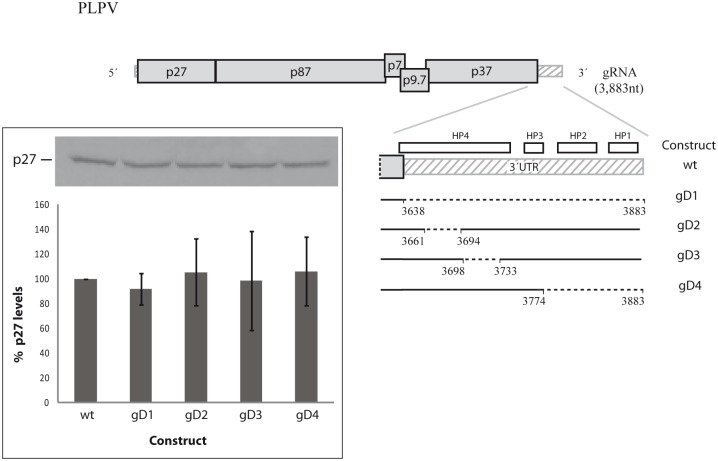
*In vitro* translation assay in WGE of wt and mutant genomic PLPV transcripts. A scheme of PLPV gRNA constructs carrying deletions (depicted as dashed lines) in the 3´-UTR is shown. Segments at the 3´-region forming potential hairpins (HP1-HP4) are indicated by small white rectangles on the wt construct. A representative autoradiograph showing p27 levels produced *in vitro* from each construct (top) and a graphic representation of such levels (bottom) are shown within an inset at the left. For the graphic representation, protein expression levels from wt gRNA were set to 100% and the translation efficiencies from other templates are indicated as percentages with respect the wt. Each percentage is shown as the mean and standard error of the mean from three replicates. Other details as in Fig 1.

To evaluate if the predicted CITE or other element of the 3´-region of PLPV genome was relevant for translation, several deletions were made in a full-length genomic construct (Fig 4). The engineered deletions led to removal of segments of the 3´-UTR encompassing nt 3661–3694 (gD2), 3698–3733 (gD3), 3774–3883 (gD4) or the entire non-coding region (nt 3638–3883, gD1). When wt and truncated viral genomic transcripts were employed as templates for *in vitro* translation reactions in WGE, the amount of *de novo* synthesized p27 was essentially maintained irrespectively of the template (Fig 4). These results suggested that no element for translation regulation was present at the 3´-UTR of PLPV or, alternatively, that if existed, it was not active in *in vitro* translation assays with WGE.

**Fig 5:**
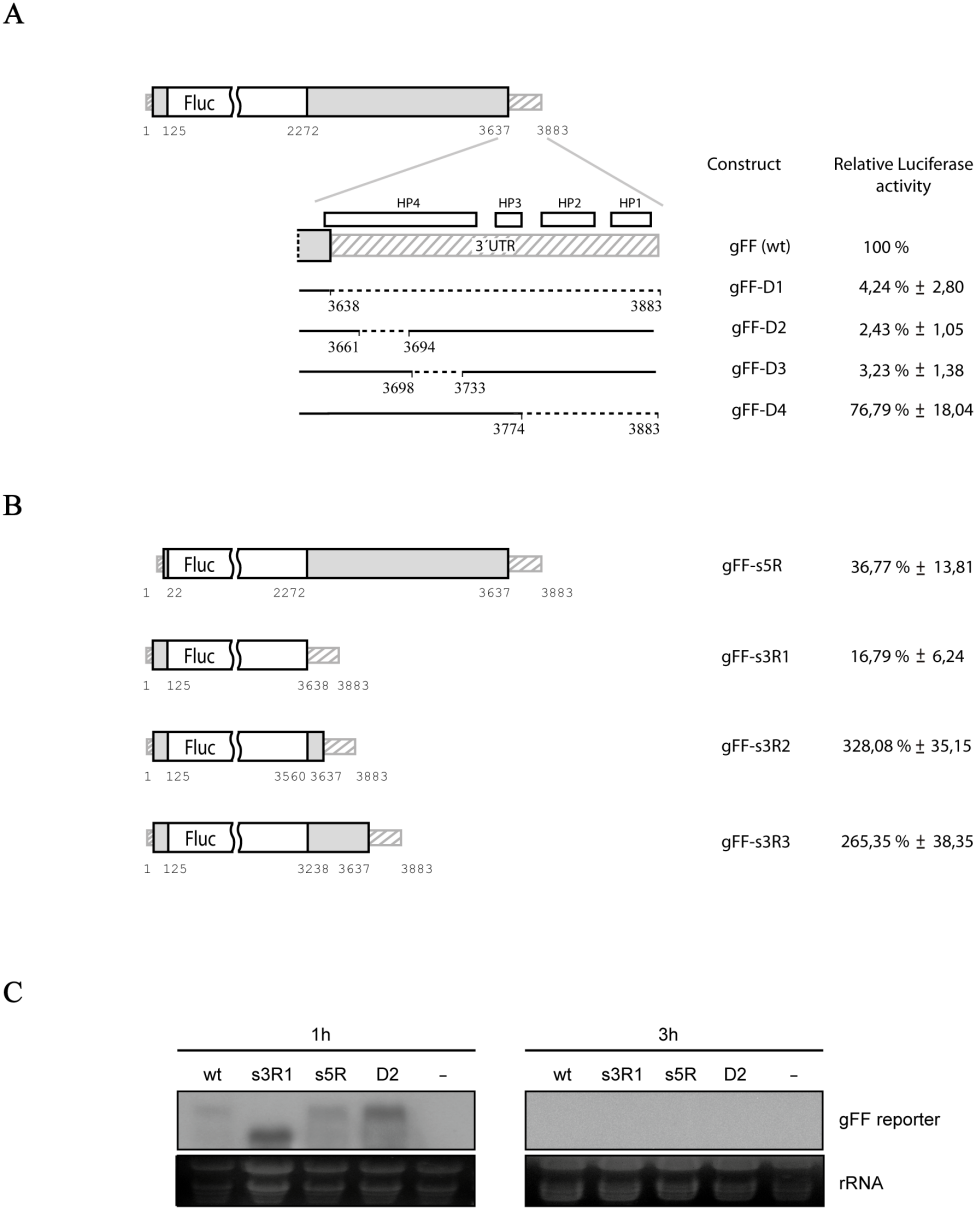
*In vivo* translation assay of reporter-based genomic PLPV transcripts. **(A)** A parental genomic PLPV construct (gFF at the top) for assessment of translation efficiency was generated by replacing most of the p27/RdRp ORF by the Fluc reporter gene (represented by a white box). This parental construct served as template for the generation of a series of mutant constructs carrying deletions at the 3´-UTR (gFF-D1 to D4). Uncapped transcripts from each construct were inoculated into *N*. *benthamiana* protoplasts along with a control capped Rluc transcript and luciferase levels were measured 3 h post-inoculation. Translation efficiencies of constructs are given as percentages with regard the parental gFF (set to 100%) The mean and standard error of the mean from three replicates are indicated. (B) gRNA reporter constructs carrying deletions at the 5´-terminal coding region (gFF-s5R) or the 3´-terminal coding region (gFF-s3R1 to s3R3). Uncapped transcripts derived from these constructs were assayed as indicated in panel A. (C) Stability of parental construct gFF and deletion constructs gFF-s3R1, gFF-s5R and gFF-D2 in protoplasts. Uncapped transcripts were employed to inoculate *N*. *benthamiana* protoplasts and total RNA was isolated at 1 h (left) and 3 h (right) after inoculation. A mock inoculated control (-) was also included. RNA was subjected to Northern blot analysis using an RNA probe derived from Fluc gene. Two replicates of the experiment were done (one shown here) with similar results. Ethidium bromide staining of ribosomal RNAs is shown below the autoradiograph as loading control. Other details as in Fig 3.

In the light of the inconclusive results with the *in vitro* approach, we decided to set up an *in vivo* translation assay for PLPV. To this end, the wt genomic construct was modified by replacing most of the RdRp gene (nt 126–2271) by the Fluc reporter gene. A series of mutants derived from this genomic reporter construct, named gFF and considered the reference wt construct, was generated by introducing deletions in the 3´- UTR as described above (Fig 5A). Uncapped transcripts synthesized from the distinct constructs were assayed in *N*. *benthamiana* protoplasts along with a control Rluc reporter RNA. Deletion of the entire 3´-UTR in construct gFF-D1 reduced translation to almost background levels, pointing to the presence of a key regulatory element in the missing region. Smaller deletions affecting the predicted HP4 (constructs gFF-D2 and gFF-D3) led to similar results whereas a deletion encompassing 3´-terminal 110 nt (and thus embracing the predicted HP1, HP2 and spacer sequence between HP2 and HP3) had only minor effects (~77% of wt levels) on translation efficiency (Fig 5A). These results were consistent with the initial prediction that HP4 corresponds to a TED-like CITE.

Additional constructs in which coding regions either downstream of the 5´-UTR (construct gFF-s5R) or upstream of 3´-UTR (construct gFF-s3R1 to R3) were removed, were also assayed for translation. The results showed that the 5´-region of 22 nt (6 nt corresponding to the 5´-UTR plus 16 nt of the p27/RdRp coding region) present in construct gFF-s5R was ~ 65% less efficient in directing translation than the 5´-region of 125 nt (6 nt of the 5´-UTR plus 119 nt of the p27/RdRp coding region) of the parental construct gFF (Fig 5B). This observation indicated that the genome segment comprised between nt 23–125 must contain a key regulatory translation element. The removal of coding sequences upstream of the 3´-UTR had disparate effects on translation efficiency (Fig 5B). The lack of viral coding sequences immediately upstream of the 3´-UTR in construct gFF-s3R1 caused a severe (~ 83%) drop in translation when compared to wt levels. This result supported that PLPV-TED corresponds to the entire HP4 (formed, as indicated above, not only by the 5´-proximal region of the 3´-UTR but also by the 3´-terminal nucleotides of p37 gene) rather than to the shorter HP (upper part of HP4; Fig 2A) previously proposed [15]. In agreement with this view, inclusion a short segment of the p37 gene (nt 3560–3637) in construct gFF-s3R2 boosted translation efficiency that was about ~ 330% with regard to gFF. An additional 5´-extension of the coding segment (nt 3238–3637) in gFF-s3R3 did not further improve translation yields but rather reduced them to 265% (Fig 5B). As construct gFF contained a 3´-coding region larger than those present in the two latter constructs but showed lower translation levels, a plausible explanation for these results was that some genome stretches down-regulate the translation process. Alternatively, the insertion of a heterologous gene (Fluc) and/or the extent of the viral sequences included in the reporter constructs might significantly affect the adoption of structures relevant for translation. Indeed, Mfold analyses revealed that HP4 is not present in the lowest free energy secondary structure of gFF, gFF-s3R2 or gFF-s3R3 though it is formed in some of their predicted suboptimal structures (data not shown). It is thus reasonable to argue that the unequal probability of HP4 formation may determine the distinct translation efficiencies of the reporter constructs.

Besides the above considerations, differences in the stability of mRNAs could indirectly affect the amount of Fluc translated. To assess whether deleting/inserting some genome regions had a significant effect on mRNA decay, some representative reporter RNAs (gFF, gFF-D2, gFF-s5R and gFF-s3R1) were selected to inoculate protoplasts and RNA levels were evaluated at 1 and 3 h post-inoculation. Northern blot analysis revealed that high and low relative translation efficiencies of the reporter RNAs could not be attributable to increased and decreased stabilities, respectively. Indeed, at 1 h after inoculation, gFF-D2 and gFF-s3R1 transcripts, both with low translation efficiencies, showed accumulation levels that were slightly higher (~ 1.7-fold and 2.2-fold, respectively) than those observed for gFF-s5R or gFF transcripts (Fig 5C, left panel). At 3 h post-inoculation, accumulation of all transcripts was negligible (Fig 5C, right panel). These results strongly supported that the relative translation efficiency of each reporter RNA is determined by presence/absence of essential structural motif(s).

### Efficient translation of a reporter-based PLPV gRNA requires establishment of a long-range RNA-RNA interaction among the TED-like CITE and a p27 ORF hairpin

**Fig 6:**
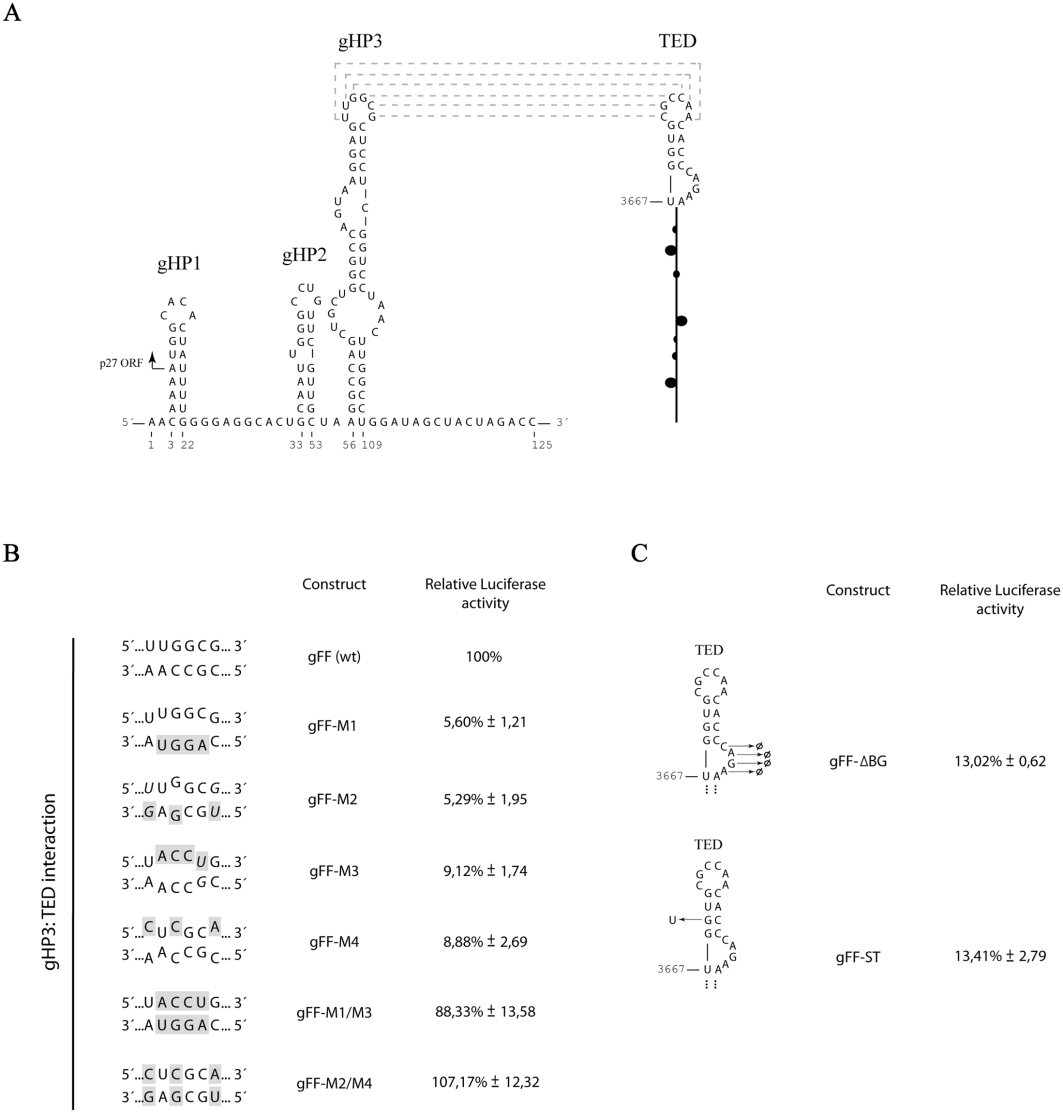
Effect of mutations in the TED-CITE and/or in a hairpin within p27 ORF on translation of reporter-based genomic PLPV transcripts. (A) Putative secondary structure of the 5´-proximal 125 nt of PLPV according Mfold predictions. A potential long-range interaction between the apical loop of a hairpin (gHP3) predicted within p27 ORF and the apical loop of the 3´-CITE is shown. Nucleotides that can putatively pair are connected by dotted lines. (B) Translation efficiency of transcripts bearing mutations that disrupted or reconstituted the potential kissing-loop interaction between apical loops of gHP3 and the CITE. All mutants were generated from parental construct gFF and assayed in *N*. *benthamiana* protoplasts as described in Fig 5 legend. Only the sequences of the loops involved in the potential base-pairing are shown. The engineered nucleotide substitutions are on a gray background and putative G:U base pairs are in italics. (C) Translation efficiency of transcripts bearing mutations in the CITE outside of its apical loop. In B and C, levels of translation as a percentage of that of gFF construct are given. Results are from three experiments with standard deviations. Other details as in Fig 5.

The deletion analysis supported the participation of the predicted TED-like CITE in PLPV gRNA translation. To further corroborate this notion, nucleotide substitutions were introduced into the apical loop of the element as its sequence was strictly conserved in TED-like CITEs predicted in other pelarspoviruses (Fig 3). Variation of the wt loop sequence, 5´-CGCCAA-3´, to either 5´-CAGGUA-3´ (gFF-M1) or to 5´-UGCGAG-3´ (gFF-M2) (nucleotide substitutions underlined) resulted in a severe drop in translation efficiency (less than 10% of that of parental construct gFF) (Fig 6) which further supported the key role of the identified CITE in the translation process and, moreover, indicated that the targeted loop has a major contribution to PLPV-TED function. As mentioned above, previous *in silico* analyses led to the suggestion that such loop could be involved in a long-range interaction, particularly with the apical loop (5´-UUGGCG-3´) of a hairpin presumably formed by a segment located within p27 ORF (gHP3 in Fig 6A) [15]. The relevance of that segment for translation was supported by the results with construct gFF-s5R that showed an important reduction in Fluc production when nt 23–125 where removed from the 5´-region of the parental construct gFF (Fig 5).

To experimentally assess whether the apical loop of gHP3 was involved in an interaction with the CITE, its sequence was changed from 5´-UUGGCG-3´ to either 5´-UACCUG-3´ (gFF-M3) or 5´-CUCGCA-3´ (gFF-M4). The translation efficiency of these new mutants, in which the predicted long-range base pairing with the CITE would be disrupted, was reduced to less than 10% of wt levels (Fig 6B), underlining the importance of mutagenized loop. Combination of the nucleotide replacements present in mutants gFF-M3 and gFF-M4 with those of the CITE mutants gFF-M1 and gFF-M2, respectively, was expected to allow reconstitution of the presumed gHP3-CITE interaction (Fig 6B). Assay of the compensatory mutants gFF-M1/M3 and gFF-M2/M4 in protoplasts revealed that, in both cases, translation efficiency was close (~ 88% for gFF-M1/M3) or even higher (~ 107% for gFF-M2/M4) than that of the wt construct. This notable recovery was consistent with the establishment of the predicted 5´-3´ long-range interaction *in vivo* and with it playing a critical role in translation.

Apart of the apical loop of the CITE (directly involved in an essential long-distance interaction as shown above), some other conserved structural features of the element were also altered to assess their contribution to functionality. Particularly, one construct in which the 4 nt upper bulge was removed (gFF-ΔBG) and another in which the 4 bp stem at the base of the apical loop was destabilized by one nucleotide replacement (gFF-ST), were generated (Fig 6C). Both engineered mutations reduced translation at similar extent (~ 13% of wt levels), highlighting the importance of the targeted traits for TED activity.

### Addition of a 5´-cap structure restores translation of CITE-deficient RNAs

**Fig 7:**
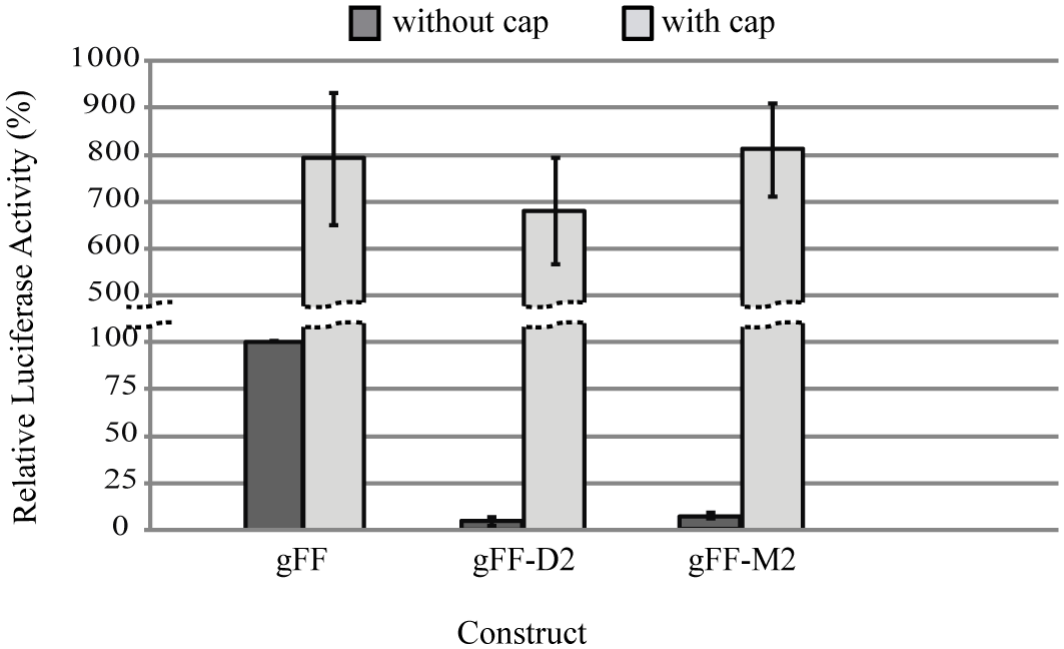
Effect of the addition of a 5´-cap on translation of reporter-based genomic PLPV transcripts. Uncapped and capped transcripts from the wt construct gFF and from CITE mutants gFF-D2 and gFF-M2 (depicted in Figs 5 and 6, respectively) were generated and their translation efficiencies estimated through assay in *N*. *benthamiana* protoplasts. Translation efficiencies of transcripts are shown as percentages with respect to that of the uncapped gFF (set to 100%) The mean and standard error of the mean from three replicates are represented.

As mentioned above, 3´-CITEs are thought to promote cap-independent translation by functionally substituting for the 5´-cap structure with high efficiency, leading to ribosome entry at or near the 5´-terminus followed by ribosome scanning to the initiation codon [13, 47, 48]. To further explore this issue in the case of PLPV-TED, we wondered whether addition of a cap could restore *in vivo* translation of CITE mutants. To this aim, we generated capped *in vitro* transcripts from parental construct gFF and from constructs gFF-D2 and gFF-M2 (Figs 5A and 6B). Measurement of Fluc activities showed that all RNAs (wt and CITE mutants) were translated with similar efficiencies (Fig 7), supporting the functional equivalence between the CITE and the cap structure. In these assays, addition of a cap to gFF transcripts increased translation by ~ 8-fold with regard uncapped gFF RNAs, which is significantly lower than the >20-fold stimulation of the cap for RNAs lacking *cis*-elements required for cap-independent translation [34]. Conversely, translation efficiencies of capped gFF-D2 and gFF-M2 were much higher (150-fold and more than 200-fold, respectively) than those of their uncapped counterparts underlining the absence of a functional CITE in the latter constructs.

### A long-range RNA-RNA interaction involving the TED-like 3´-CITE and a hairpin of the 5´-UTR is also indispensable for efficient translation of a reporter-based PLPV sgRNA

**Fig 8:**
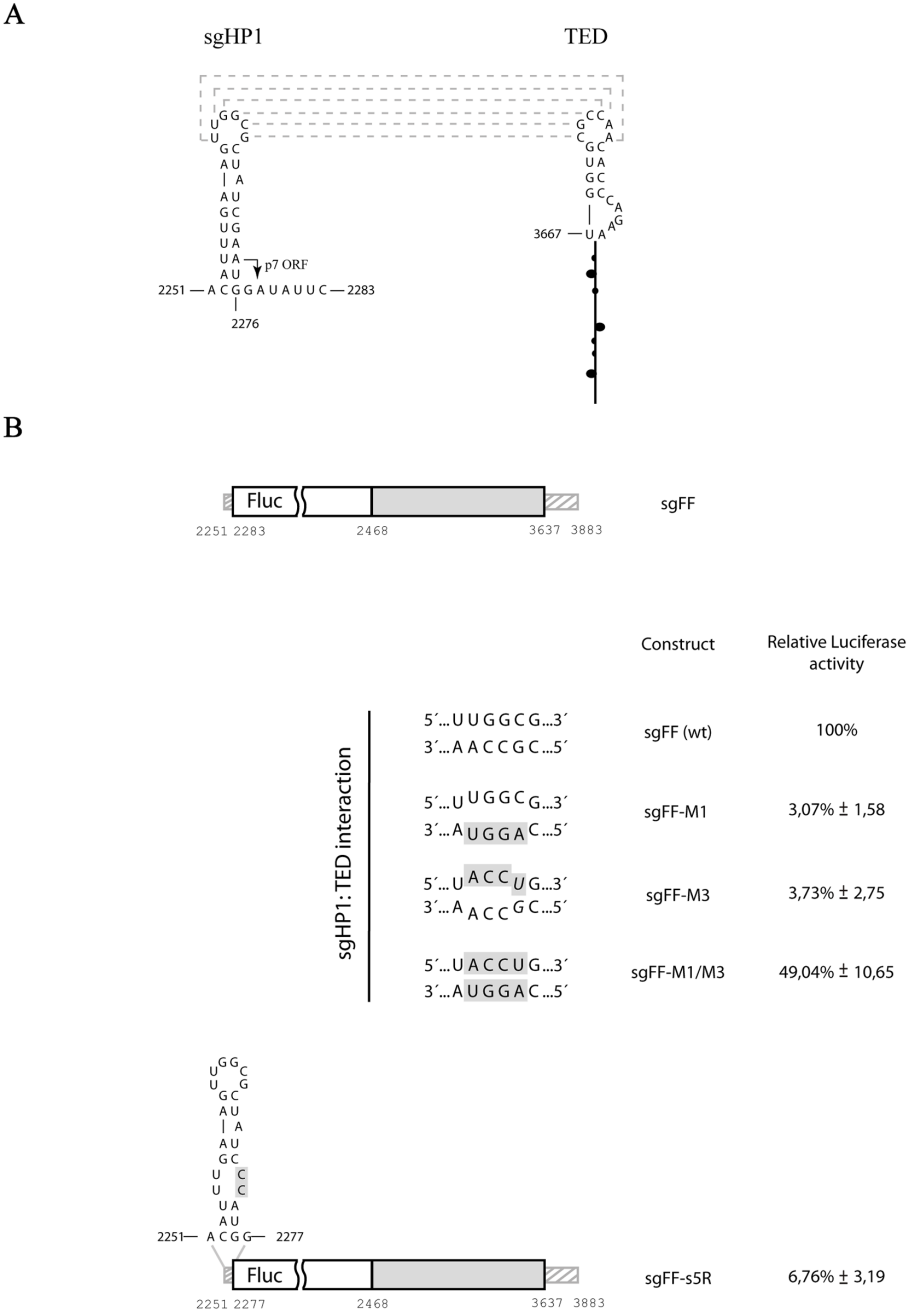
Assessment of the relevance of the 3´-TED and/or a 5´-hairpin, that can potentially establish a kissing-loop interaction, on reporter-based PLPV subgenomic translation. A) Putative secondary structure of the 5´-proximal 33 nt of PLPV sgRNA according Mfold predictions. The start codon of p7 ORF is indicated by an arrow. A potential long-range interaction between the apical loop of a 5´-hairpin (sgHP1) and the apical loop of the TED-like CITE is shown. Nucleotides that can putatively pair are connected by dotted lines. (B) *In vivo* translation assay of subgenomic PLPV transcripts. A parental subgenomic construct for assessment of translation efficiency was generated by replacing almost the entire p7 ORF by the Fluc reporter gene. This parental construct, named sgFF, is shown at the top and mutants derived from it are depicted below. One of the mutants had a shorter 5´-region (sgFF-s5R) and the others carried mutations that disrupted (sgFF-M1 and M3) or preserved (sgFF-M1/M3) the potential kissing-loop interaction between the 3´-CITE and the 5´-sgHP1. For the latter mutants, only the sequences of the loops involved in the potential base-pairing are shown. The engineered nucleotide substitutions are on a gray background and putative G:U base pairs in potential kissing-loop interaction(s) are in italics. Translation efficiencies were measured in protoplasts and the mean and standard error of the mean from three replicates is shown. Other details as in Fig 5.

The above results with genomic constructs supported that communication of the CITE with the 5´-end is aided by a long-distance RNA-RNA interaction. A similar interaction could be predicted in the sgRNA, an uncapped viral RNA species whose translation was expected to rely also on the CITE. The elements directly involved in the potential interaction would be the apical loop of the CITE and the apical loop of a hairpin (sgHP1) formed by the 5´-UTR plus the start codon of p7 ORF (Fig 8A). The sequence of the apical loop of sgHP1 is identical to that of gHP3 and, thus, the putative kissing-loop interaction in the sgRNA would be formed by the same base pairs than that of the gRNA.

To evaluate *in vivo* translation of the sgRNA, the almost entire p7 ORF (nt 2284–2467) was replaced by the Fluc reporter gene giving rise to the parental construct sgFF (Fig 8B) from which efficient translation was recorded in *N*. *benthamiana* protoplasts. Alteration of the apical loop of the CITE from 5´-CGCCAA-3´ to 5´-CAGGUA-3´ in construct sgFF-M1, which would abrogate the presumed kissing-loop interaction with sgHP1, diminished translation to less than 5% of wt levels (Fig 8B). These results were essentially identical to those obtained with the genomic construct carrying the same alteration (gFF-M1, Fig 6B) and confirmed the functionality of CITE in the sgRNA context. Introduction of disrupting mutations in the apical loop of sgHP1, particularly change of 5´-UUGGCG-3´ to 5´-UACCUG-3´ (construct sgFF-M3, Fig 8B), had a similar effect as translation was decreased to ~ 4% of wt levels. Combination of the nucleotide substitutions of both mutants, which were compensatory regarding the predicted kissing-loop interaction, in construct sgFF-M1/M3 raised translation to 50% of wt levels. The results nicely supported that, as observed in the reporter-based gRNA, a long-range interaction among the 3´-CITE and the 5´-region is needed for efficient sgRNA translation. Formation of the entire sgHP1 was apparently required for the establishment of such interaction as the translation of construct sgFF-s5R, with a shortened 5´-region (nt 2251–2277) and with the formation of the lower stem of sgHP1 impaired (as a consequence of the introduction of an *Nco*I restriction site to insert the Fluc gene), was reduced to ~ 14% of wt levels.

### Preservation of translationally relevant 5´-3´ communication is critical for PLPV infectivity

**Fig 9:**
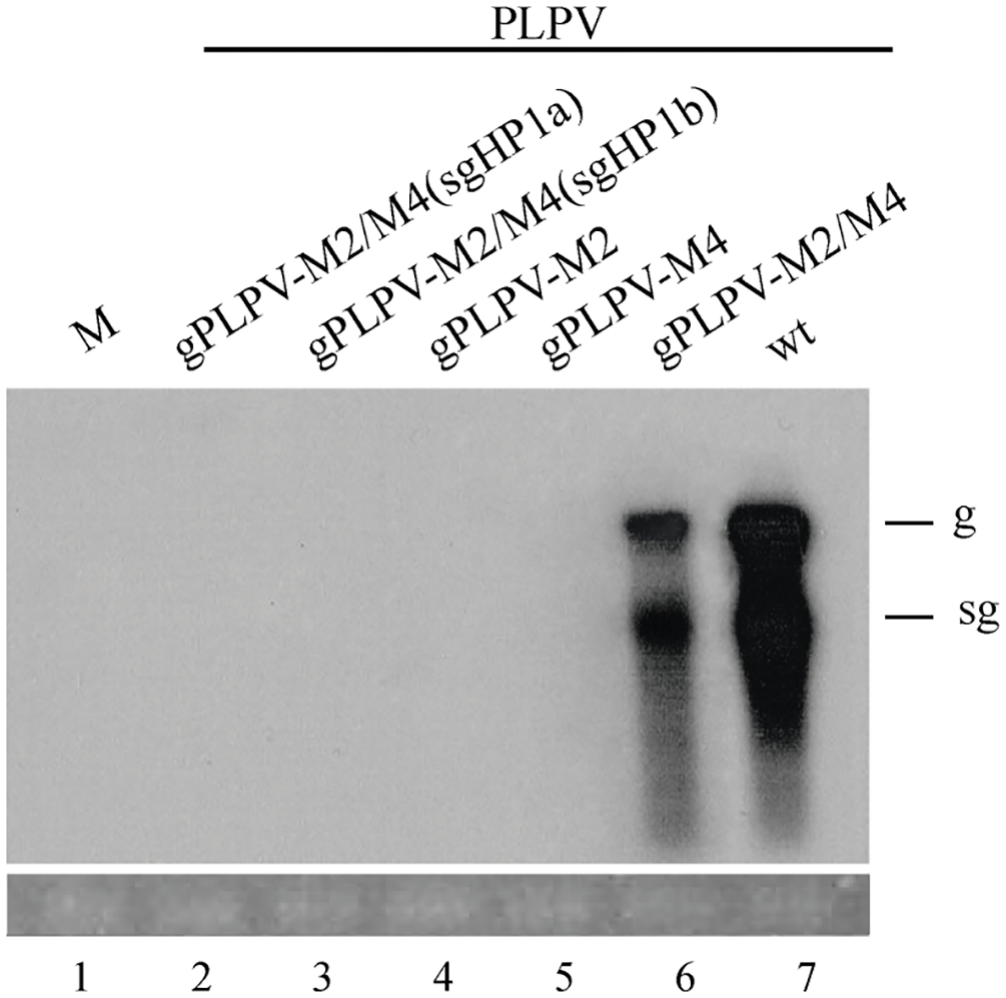
Assessment of infectivity of PLPV mutants. Uncapped transcripts corresponding to the gRNA of wt and CITE-related mutants of PLPV were inoculated onto *N*. *benthamiana* plants. Local leaves were collected at 15 days post-inoculation and viral accumulation was determined by Northern blot analysis. Bioassayed mutants are indicated above the lanes of the autoradiograph. A mock inoculated sample (lane M) was also included. Positions of PLPV gRNA and sgRNA are indicated at the right. Ethidium bromide staining of ribosomal RNAs is shown below the autoradiograph as loading control.

Our assays in protoplasts with PLPV reporter constructs clearly highlighted the importance of a 5´-region/3´-CITE communication for efficient translation. In order to corroborate the key role of such communication in the context of infection, *N*. *benthamiana* plants were inoculated with uncapped transcripts from PLPV constructs containing mutations that either disrupted (gPLPV-M2 and M4, with nucleotide replacements in the apical loop of the CITE and gHP3, respectively) or restored (gPLPV-M2/M4) the gHP3-CITE kissing-loop interaction (Table 1). We choose these specific mutations because they had been tested in translation assays with the corresponding reporter constructs (gFF-M2, gFF-M4 and gFF-M2/M4; Fig 6) and, also, because those affecting gHP3 did not cause alterations at amino acid level (i.e., the p27/RdRp sequences of the mutants were identical to those of the wt virus). Northern blot analysis of the inoculated plants revealed than neither mutant gPLPV-M2 nor -M4 were able to establish infections (lanes 4 and 5 in Fig 9). Conversely, viral RNAs were detected in all plants inoculated with mutant gPLPV-M2/M4 (lane 6 in Fig 9) indicating that the maintenance of the long-range interaction between the 5´-region and the 3´-CITE is a prerequisite for viral infectivity. Nevertheless, mutant gPLPV-M2/M4 accumulated in inoculated leaves at levels considerably lower than those of the wt virus (lane 7 in Fig 9) and, most likely for this reason, failed to systemically invade the plants. This mutant did not present changes in the encoded proteins and, moreover, the usage frequencies of the mutated codons *vs* the wt ones, a factor that may influence protein production [49], were very similar for the RdRp gene (data not shown). We reasoned that the lower accumulation of the mutant could either be due to the participation of the mutagenized regions in processes other than translation or, more likely, to the disruption of the interaction among the 3´-CITE and sgHP1, as the nucleotide substitutions in the former were not compensated in the latter (Table 1). To alleviate this lack of compensation, two additional viral mutants were generated using construct gPLPV-M2/M4 as template: mutants gPLPV-M2/M4-sgHPa and -sgHPb, in which the apical loop of sgHP1 was changed from 5´-UUGGCG-3´ to 5´-CUCGCA-3´ and 5´-UUCGCG-3´, respectively. In the first case, the sgHP1-CITE interaction would be formed by canonical G:C and A:U base pairs, as in the wt virus, whereas in the second construct such interaction would contain two wobble G:U base pairs (Table 1). Bioassay of the new constructs showed that none of them was capable of infecting *N*. *benthamiana* plants (lanes 2 and 3 in Fig 9). Since the mutations introduced in sgHP1 to reconstitute the interaction with the CITE led to one (construct gPLPV-M2/M4-sgHP1b) or three (construct gPLPV-M2/M4-sgHP1a) amino acid replacements at the C-terminus of the RdRp (Table 1), the possibility that these replacements were lethal to the virus seemed very plausible.

**Table 1:** Bioassay of PLPV (wt and mutants) in *N*. *benthamiana* plants.

Virus variant	gHP3:CITEinteraction^a^	sgHP1:CITEinteraction	p87aa sequence(nt sequence)	No. infected plants/No. inoculated plants^b^	Viral accumulation(%)^c^
gPLPVwt	5´…UUGGCG…3´3´…AACCGC…5´	5´…UUGGCG…3´3´…AACCGC…5´	wt	6/6	100
gPLPV-M2	5´…*U*UGGC*G*…3´3´…*G*AGCG*U*…5´	5´…*U*UGGC*G*…3´3´…*G*AGCG*U*…5´	wt	0/4	-
gPLPV-M4	5´…CUCGCA…3´3´…AACCGC…5´	5´…UUGGCG…3´3´…AACCGC…5´	wt	0/4	-
gPLPV-M2/M4	5´…CUCGCA…3´3´…GAGCGU…5´	5´…*U*UGGC*G*…3´3´…*G*AGCG*U*…5´	wt	6/6	15,2 ± 5,8
gPLPV-M2/M4 (sgHP1a)	5´…CUCGCA…3´3´…GAGCGU…5´	5´…CUCGCA…3´3´…GAGCGU…5´	V752A (T2261C)G753R G2263C)A754T (G2266A)	0/6	-
gPLPV-M2/M4 (sgHP1b)	5´…CUCGCA…3´3´…GAGCGU…5´	5´…*U*UCGC*G*…3´3´…*G*AGCG*U*…5´	G753R (G2263C)	0/6	-

^a^ Engineered mutations underlined; G:U base pairs in italics

^b^Data regarding inoculated leaves since mutant gPLPV-M2/M4 did not systemically infect plants.

^c^ Assessment of viral accumulation was performed by Northern blot analysis. Viral accumulation of wt virus was set to 100% and the mean accumulation of the mutant virus and standard deviation of the mean as estimated from independent plants are given

In the light of the above results, we decided to explore whether evolution of mutant gPLPV-M2/M4 could lead to accumulation of changes in the virus genome that tended to stabilize the sgHP1-CITE interaction. To this aim, this construct was inoculated into *C*. *quinoa* plants and the viral progeny was subjected to serial passages in the same plant species. We chose this local host because studies with related viruses suggested it promotes quick virus evolution [50, 51]. In agreement with the results in *N*. *benthamiana* plants (Table 1), appearance of lesions on *C*. *quinoa* leaves inoculated with mutant gPLPV-M2/M4 was delayed 3–5 days with regard to that observed with the wt virus (inoculated as a control in parallel assay) and, also, the number of lesions caused by the former was notably lower than that recorded with the latter. After six serial passages, the relative timing of appearance and number of lesions were essentially maintained between both virus lineages. RT-PCR amplification and sequencing of gPLPV-M2/M4 progeny at the third to sixth passages showed that the original mutations in gHP3 and in the CITE were maintained in all cases and that no additional mutations were present within sgHP1. The lack of compensatory mutations in sgHP1 that could favour its interaction with the mutated CITE probably reflects the strong selection pressure that imposes the coding nature of the sequences involved in formation of sgHP1. On its side, variations in the CITE enhancing its base-pairing with sgHP1 are neither likely tolerated because they might impair interaction of the element with gHP3 or drive variations in the latter that could result in p27/RdRp amino acid changes incompatible with protein function.

## Discussion

**Fig 10:**
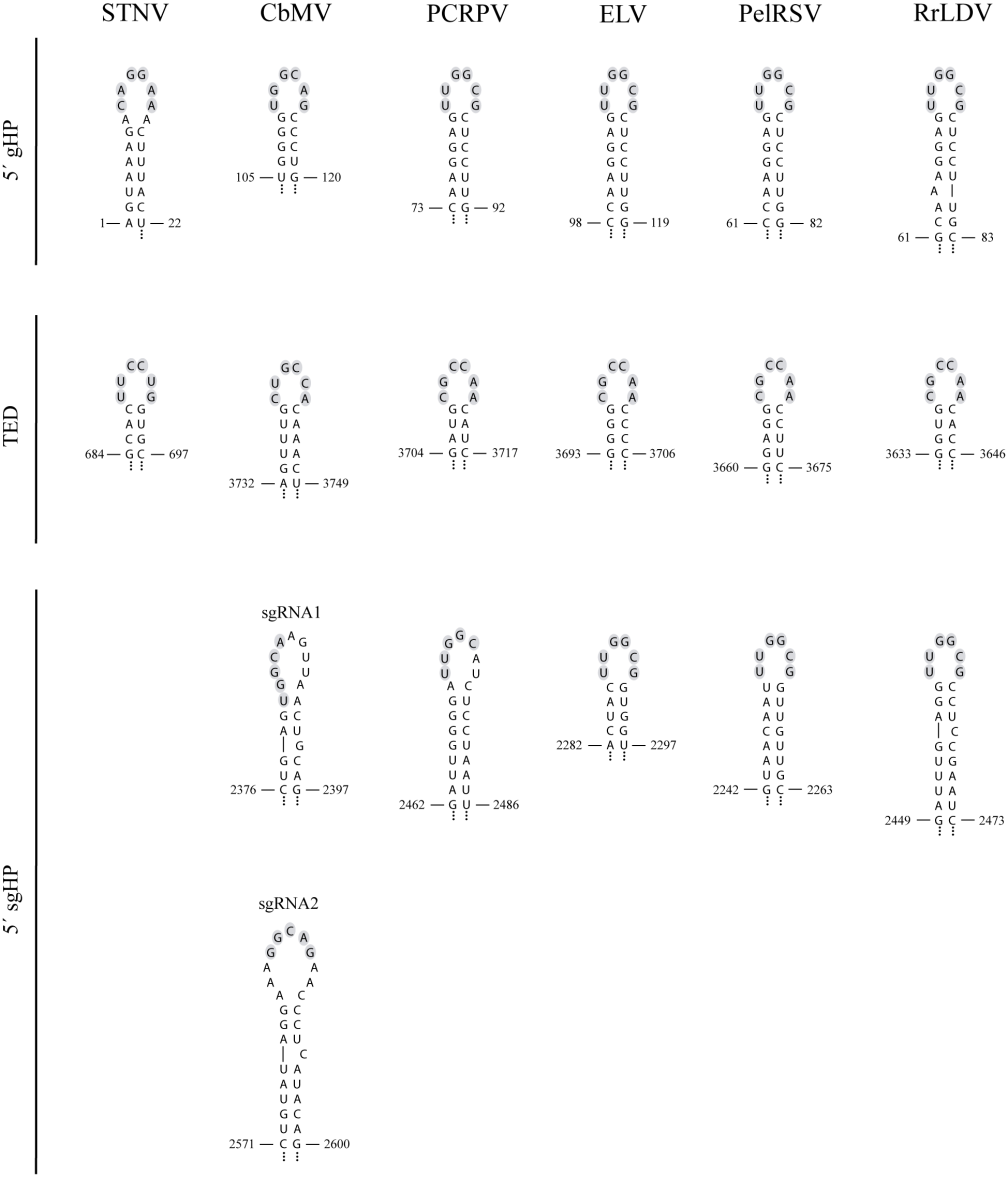
STNV-TED and putative TED-like CITEs predicted in the 3´ UTR of several viruses (middle panel) can potentially base-pair with the 5´-region in the gRNA (upper panel) or sgRNA (lower panel) context. Virus acronyms as is Fig 3.

In the present work we have confirmed the functionality of a TED-like CITE predicted in the 3´-region of PLPV. Using a combination of *in silico*, *in vitro* and *in vivo* approaches, we have also provided firm evidence that this element is larger than previously anticipated [15] and we have obtained data supporting that STNV-TED and other presumed TED-like CITEs most likely adopt similar extended conformations. In addition, the results have revealed that the establishment of a long-range RNA-RNA interaction between PLPV-TED and the 5´-region of the viral RNA is imperative for efficient translation. Interaction of the putative TED-like CITE of CbMV or of PCRPV with a 5´-terminal genomic hairpin has been also predicted [15] and similar interactions can be anticipated for ELV, PelRSV and RrLDV (Fig 10), suggesting that the activity of all of them might rely on communication through base-pairing with the 5´-terminus. Long-range RNA-RNA interactions have been proposed to mediate the activity of essentially all types of CITEs but the T-shaped class [52]. However, formal demonstration of such issue has been achieved only for a limited number of CITE-containing viruses [39, 44, 47, 48, 53, 54] and, as indicated before, it remains to be accomplished for STNV-TED. In the light of the results obtained with PLPV-TED, the relevance of potential interactions between STNV-TED and 5´-proximal sequences of the RNA would deserve to be subjected to a thorough reassessment.

The mutagenesis compensatory approach performed here has clearly revealed that gHP3 contains the genomic 5´-partner of the CITE, as recently proposed [15]. Two different set of mutations designed to re-establish disrupted base-pairing between the CITE and gHP3 (implying three -construct gFF-M2/M4- or four–construct gFF-M1/M3- nucleotide replacements in each element) were very effective at restoring cap-independent translation. These results suggested that the primary sequences of the apical loops directly involved in the interaction are not relevant for translation and that the main role of such loops in the translation process is to promote contact among the 5´- and the 3´- regions of the gRNA. In this sense, the high sequence conservation of the interacting loops in CITEs (or presumed CITEs) of different classes (TED, PTE, I-shaped and T-shaped) of carmo-, umbra- and pelarspoviruses [15] is puzzling and, even though other explanations cannot be discarded, it could be conditioned by the involvement of the interacting stretches in function(s) other than translation.

PLPV-TED has been found to be active in both, the gRNA and the sgRNA reporter contexts. This situation parallels that reported in other *Tombusviridae* that contain distinct CITE classes and that, like PLPV, produce sgRNA(s) during infection [47, 54–56]. Moreover, despite the precise 5´-ends of the sgRNAs generated by other pelarspoviruses have not been mapped, complementarity between the TED-like CITE and a sequence located close to the initiation codon of p7 gene can be detected in all cases (Fig 10). In a similar line, complementarity between the apical loop of the potential TED-like CITE of CbMV and the apical loop of a HP structure predicted in the 5´-region of either the sgRNA1 or the sgRNA2 of this virus, can be found (Fig 10). Thus, *in silico* analysis would support the functionality of the predicted TED-like CITEs in the distinct RNAs produced by each virus.

In addition to highlighting the relevance of the apical loop of both the CITE and 5´-terminal hairpins (gHP3 and sgHP1), the results have shown that the 3´-lateral bulge of the former is also essential to preserve the translational activity of the element. Most likely, such lateral bulge induces bending of the CITE structure, enhancing its flexibility and favouring its interaction with the 5´-partner. Alternatively, the bulge might direct binding of essential factor(s), since such type of motif has been identified as recognition site for proteins in different viral and non-viral RNAs [57]. Bulges similar to that of PLPV-TED are present in STNV-TED and in the TED-like CITEs predicted in CbMV and in the other pelarspoviruses (Fig 3), a phylogenetic conservation that further supports their functional significance. The maintenance of the 4 bp helix at the base of the apical loop of PLPV-TED, another conserved secondary structure trait (Fig 3), seemed also critical for CITE function. Further mutagenesis of the CITE will help to define further details on its structural requirements.

The bioassay of PLPV mutants confirmed that maintenance of the 5´-3´ gRNA communication is critical for virus infectivity. In addition, the absence of mutations in either sgHP1 or the CITE of gPLPV-M2/M4 progenies after serial passages suggested that the coding nature of the CITE 5´-partner(s) must impose important selective pressures on population genetic structure. Compensatory mutations in sgHP1 would lead to amino acid change(s) with detrimental effects on RdRp function whereas sequence modifications in the CITE would most likely force alterations in gHP3 leading to amino acid replacement(s) in p27/p87 that might severely impair their activity. The notably reduced fitness (with regard the wt virus) of gPLPV-M2/M4 and progenies in plant bioassays is consistent with an inefficient interaction between sgHP1 and the CITE that must result in low sgRNA translation levels.

To conclude, the results presented here have provided important new insights into the structural requirements of a TED-like CITE, one of the least studied CITE classes, and into its interacting partners within the viral genome. Evidence in support of a key role of the CITE-mediated translation for viral infectivity has also been obtained. The apparent lack of functionality of PLPV-TED in *in vitro* assays, which parallels that found for the Y-shaped CITE of TBSV or the T-shaped CITE of *Turnip crinkle virus* [52, 58], has precluded performance of binding assays with WGE (or with purified wheat eIFs) in order to identify factors recruited by this regulatory element. Alternative approaches are being developed to tackle this question that may substantially improve our knowledge on the mechanistic bases of the PLPV CITE.
